# Induced pluripotent stem cell-based therapies for organ fibrosis

**DOI:** 10.3389/fbioe.2023.1119606

**Published:** 2023-05-18

**Authors:** Wei Cheng, Chengming Fan, Qing Song, Ping Chen, Hong Peng, Ling Lin, Cong Liu, Bin Wang, Zijing Zhou

**Affiliations:** ^1^ Department of Pulmonary and Critical Care Medicine, Second Xiangya Hospital, Central South University, Changsha, China; ^2^ Research Unit of Respiratory Disease, Central South University, Changsha, China; ^3^ Department of Cardiovascular Surgery, Second Xiangya Hospital, Central South University, Changsha, China; ^4^ Department of Thoracic Surgery, Second Xiangya Hospital, Central South University, Changsha, China

**Keywords:** induced pluripotent stem cells (iPSC), stem cell, fibrosis, cell therapy, human induced pluripotent stem cells (hiPSC)

## Abstract

Fibrotic diseases result in organ remodelling and dysfunctional failure and account for one-third of all deaths worldwide. There are no ideal treatments that can halt or reverse progressive organ fibrosis, moreover, organ transplantation is complicated by problems with a limited supply of donor organs and graft rejection. The development of new approaches, especially induced pluripotent stem cell (iPSC)-based therapy, is becoming a hot topic due to their ability to self-renew and differentiate into different cell types that may replace the fibrotic organs. In the past decade, studies have differentiated iPSCs into fibrosis-relevant cell types which were demonstrated to have anti-fibrotic effects that may have the potential to inform new effective precision treatments for organ-specific fibrosis. In this review, we summarize the potential of iPSC-based cellular approaches as therapeutic avenues for treating organ fibrosis, the advantages and disadvantages of iPSCs compared with other types of stem cell-based therapies, as well as the challenges and future outlook in this field.

## 1 Background

Fibrosis is mainly characterized by parenchymal cell destruction, local activation of fibroblasts, and excessive accumulation of extracellular matrix (ECM) which results in loss of normal architecture and function of nearly all major organs including lung, heart, liver, skin, and kidney (Zeisberg and Kalluri, 2013; Nanthakumar et al., 2015; Vijayaraj et al., 2019). In the early stages, fibrosis is thought to be reversible, but with disease progression, it can lead to organ dysfunction and death (Wynn and Ramalingam, 2012; Zeisberg and Kalluri, 2013). Since there are still no effective treatments that can halt or reverse this pathological process, stem cell-based therapy seems to bring new hopes for patients with fibrotic diseases due to their abilities to self-renew and differentiate into different cell types that may replace or regenerate the dysfunctional organ (Blanpain and Fuchs, 2014; Vijayaraj et al., 2019; Cao et al., 2020).

Pluripotent stem cells (PSCs) include induced pluripotent stem cells (iPSCs) and embryonic stem cells (ESCs). The above cells can self-renew and differentiate into ectoderm, mesoderm, and endoderm derivatives. (Dvash et al., 2007; Takahashi et al., 2007). iPSCs are pluripotent stem cells that are produced by the gene transfer of reprogramming factors to human somatic cells, which was first developed by Shinya Yamanaka and his team in 2006 (Takahashi and Yamanaka, 2006). In 2013, Yamanaka won the Nobel Prize in Physiology or Medicine for revealing that adult cells can be reprogrammed. The pluripotency of iPSCs is induced by a combination of reprogramming factors such as OCT3/4, SOX2, KLF4, c-MYC, and LIN28 (Okita et al., 2013; Parrotta et al., 2020). Because iPSCs are readily available, suitable for mass production, and eliminate immune rejection, iPSCs could be a promising alternative to ESCs (Fan et al., 2020).

Induced pluripotent stem cell (iPSC) is thought to be an important resource in regenerative medicine because of their pluripotency and self-renewal ability (Kamiya et al., 2019). Since the development of iPSC technology (Takahashi and Yamanaka, 2006), iPSCs have become one of the most promising sources of stem cells in the cell therapy application of organ fibrosis (Alvarez-Palomo et al., 2020; Fan et al., 2020; Bian et al., 2021; Nuciforo and Heim, 2021). As previously reported, iPSCs and iPSCs-derived epithelial cells, macrophages, cardiomyocytes (CMs), and endothelial cells had a potential influence in repairing damaged tissue in organs and tissue fibrosis (Dai et al., 2011; How et al., 2013; Alvarez-Palomo et al., 2020; Pouyanfard et al., 2021). For instance, the transplantation of iPSCs or iPSCs-derived alveolar epithelial cells could reduce lung fibrosis in pulmonary fibrosis (PF) model mouse (How et al., 2013; Alvarez-Palomo et al., 2020). And the iPSCs-derived macrophage populations could reduce fibrogenic gene expression and disease-associated histological markers in liver fibrosis model mouse (Pouyanfard et al., 2021). What’s more, iPSCs significantly improve the potential of developing personalized cell-based therapy for diseases like cystic fibrosis, since iPSCs can differentiate into patient-specific stem cells and repair tissues damaged by disease pathology (Suzuki et al., 2016).

**FIGURE 1:**
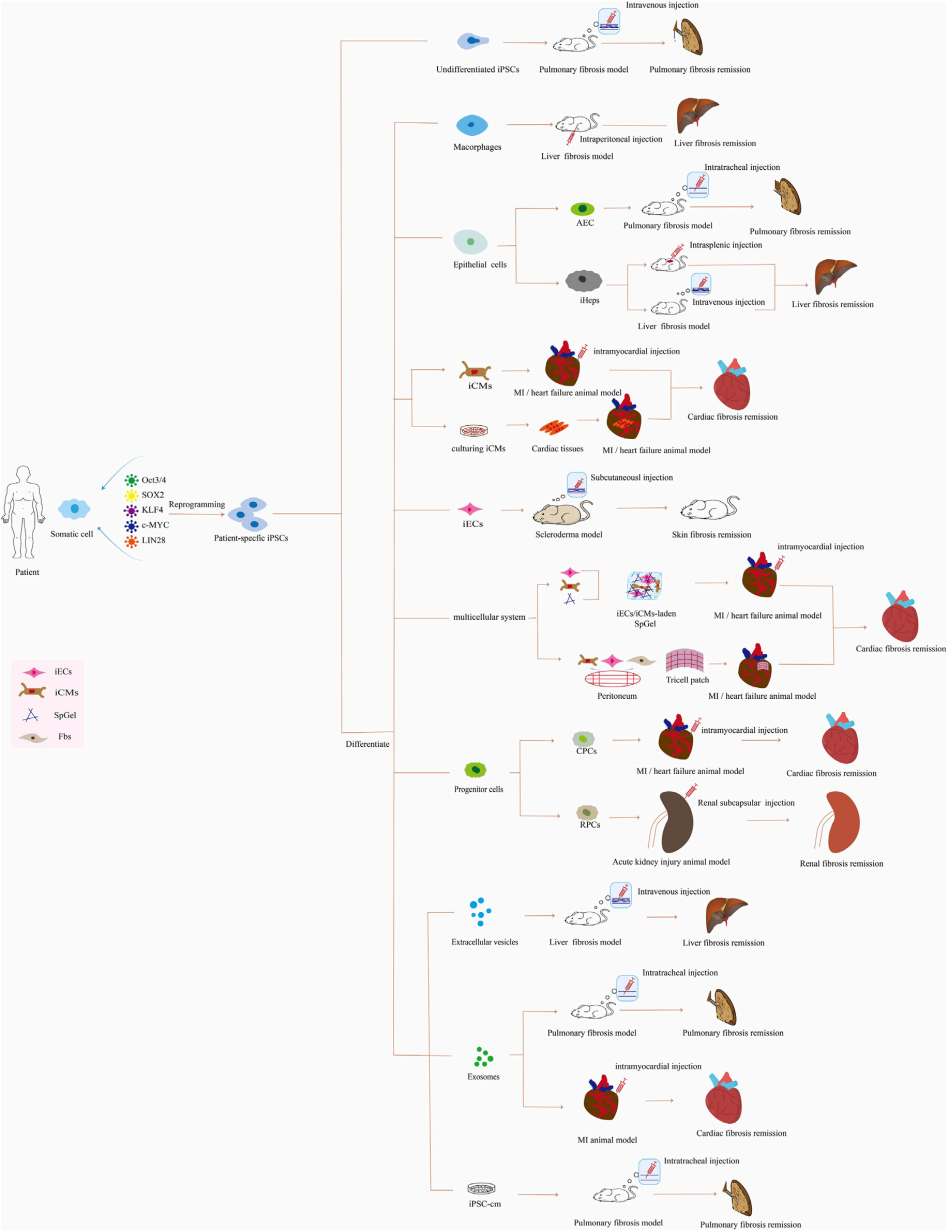
Application of patient-specific iPSC-based therapies for organ fibrosis *in vivo*. Abbreviations: iPSCs, induced pluripotent stem cells; AEC, alveolar epithelial cells; iHeps, iPSCs-derived hepatocyte; iCMs, iPSC-derived cardiomyocyte; MI, myocardial infarction; iECs, iPSC-derived endothelial cell; SpGel, thermoresponsive hydrogel; Fbs, fibroblasts; CPCs, cardiac progenitor cells; RPCs, renal progenitor cells; iPSC-cm, the secretome of induced pluripotent stem cell.

In this review, we mainly summarize the iPSC-based therapeutic approaches for treating organ fibrosis (Figure 1; Supplementary Table S1), the advantages and disadvantages of iPSCs compared with other therapies, and the challenges and future outlook in this field.

## 2 iPSC-based therapy for organ fibrosis

### 2.1 iPSCs and iPSCs-derived cell-based therapy for organ fibrosis

#### 2.1.1 iPSCs

iPSCs characterized by pluripotency and ultimate differentiation into any disease-related cell type make this a reliable avenue toward studying organ fibrosis. As previously reported, the intravenous administration of iPSCs markedly inhibited bleomycin-mediated activation of TGF-β1/Smad2/3 and epithelial cell to mesenchymal transition (EMT) by up-regulating the epithelial marker E-cadherin and down-regulating mesenchymal markers including fibronectin, vimentin and α-SMA in PF model mouse (Zhou et al., 2016). Nevertheless, Zhou et al. (2014) found intratracheally transplantation of undifferentiated iPSCs did not reduce lung fibrosis in the bleomycin-induced mouse model. Another study demonstrated that intravenous delivery of iPSCs lacking the reprogramming factor c-MYC (three-gene iPSCs) could alleviate the severity of lung fibrosis by reducing collagen content and myeloperoxidase activity, diminished neutrophil accumulation in bleomycin-induced PF model mouse (How et al., 2013). The above studies indicate that the effect of transplantation of undifferentiated iPSCs on the remission of bleomycin-induced lung fibrosis in mouse is still controversial, and these difference in research results may be related to the way of transplantation.

#### 2.1.2 Macrophages

Fibrosis is a complex disease that involves multiple cell types including macrophages (Bataller and Brenner, 2005; Pan et al., 2021). Evidence showed that the induction of the “restorative phenotype” of macrophages could alleviate the progression of fibrosis by establishing an anti-inflammatory microenvironment (Weiskirchen et al., 2019). Therefore, macrophages have been proposed as potential targets for the prevention or treatment of tissue fibrosis (Pechkovsky et al., 2010; Pouyanfard et al., 2021). Indeed, Somayeh et al. demonstrated that human iPSCs-derived macrophage populations, especially the M2 subtype, could reduce fibrogenic gene expression and disease-associated histological markers including sirius red, α-smooth muscle actin (α-SMA) and desmin in an immunodeficient mouse liver fibrosis model, making this approach a promising cell-based avenue to ameliorate fibrosis (Pouyanfard et al., 2021). Liver Kupffer cells (KCs) were a type of heterogeneous macrophage lineage cells (Kinoshita et al., 2010). Tasnim et al. (2019) differentiated MYB-independent iPSCs into macrophage precursors and exposed them to hepatic cues to generate iPSCs-derived KCs (iKCs) which were mature, liver-specific, and functional. Finally, they showed that iKCs offer a mature renewable human cell source for liver-specific macrophages, useful in developing to ameliorate fibrosis.

#### 2.1.3 Alveolar epithelial cells

The injury of alveolar epithelial cells, the failure of proper healing or regeneration, and the process of EMT are central to the pathogenesis of PF (King et al., 2011; Alvarez-Palomo et al., 2020). Yan et al. (2014) showed that human iPSCs-derived alveolar type II cells were able to efficiently remain and re-epithelialize injured alveoli to restore pulmonary function and prevent lung fibrosis. Furthermore, Alvarez-Palomo et al. (2020) confirmed that endotracheal transplantation of human iPSCs-derived alveolar epithelial type 2 cells (AEC2) could reduce lung fibrosis when PF already occurs and reduces the amount of collagen by inhibiting TGF-β and α-SMA expression in PF model mouse. Zhou et al. (2014) also found that intratracheally transplantation of mouse iPSCs-derived AEC2 could attenuate lung fibrosis and reduce lung inflammation in bleomycin-induced model mouse. These above studies suggest that iPSCs-derived-AEC2 can be used to treat lung fibrosis by replacing abnormal or impaired AEC2.

#### 2.1.4 Hepatocytes

Liver fibrosis is caused by chronic liver injury and its pathogenesis is closely related to hepatocyte death which may result in abnormal scar tissue in the injured liver (Bataller and Brenner, 2005; Wang et al., 2016). Since damaged hepatocytes and complex interactions between these different hepatic cells mainly contributed to the pathogenesis of liver fibrosis, scientists transplanted these different hepatic cells generated from iPSCs into fibrotic livers and found a significant anti-fibrotic property. Studies revealed that engraftment of iPSCs-derived hepatocytes (iHeps) or hepatocyte-like cells (HLCs) significantly decreased collagen content, level of pro-inflammatory factors, increased number of proliferating hepatocytes and rescued liver function detected by concentrations of ALT, AST, ammonia, and total bilirubin in the serum of mouse liver fibrosis model (Takayama et al., 2017; Choi et al., 2020). The above researches are mainly to alleviate fibrosis by inactivating inflammatory pathways or establishing an anti-inflammatory microenvironment and reducing ECM protein. Furthermore, Wei et al. (2022) found that transplantation of genetically corrected patient-specific iHeps by using CRISPR/Cas9 into immunodeficient Wilson’s disease (WD) mouse model can rescue the function of ATPase copper transporting beta (ATP7B) and disease phenotypes of WD characterized by improvement in liver function, reduced liver fibrosis and decreased hepatic copper accumulation due to copper-induced hepatotoxicity. These researches indicate that iHeps and or genetically corrected iHeps may be used to rescue liver fibrosis and liver function by replacing abnormal or impaired iHeps.

#### 2.1.5 Cardiomyocytes

Myocardial fibrosis is characterized by increased ECM and increased fibroblast numbers resulting from myocardial ischemia, finally leading to the loss of functional CMs (Nielsen et al., 2019). The transplantation of iPSCs-derived CMs (iPSCs-CMs) could reduce myocardial fibrosis, but the underlying mechanism of iPSCs-CMs-based cell therapy is still uncertain (Jung et al., 2017). Several studies have shown that transplant patient iPSCs-derived CMs (iCMs) can attenuate cardiac fibrosis in myocardial infarction (MI) *in vivo* models (Singla et al., 2011; Citro et al., 2014; Fang et al., 2020; Stępniewski et al., 2020; Suzuki et al., 2021). For example, the transplantation of human iCMs as cardiac tissue into the hearts improved cardiac function by alleviating interstitial fibrosis, decreasing hypertrophied CMs, and increasing capillary density in a porcine cardiomyopathy model (Suzuki et al., 2021). And Fang et al. (2020) revealed that rats receiving iPSCs-CMs transplantation via intramyocardial injection could partially rescue the injured cardiac region, and reduced cardiac fibrosis in the heart failure rat. Furthermore, Stępniewski et al. (2020) demonstrated that the transplantation of iCMs or iCMs overexpressing either the proangiogenic SDF-1α or anti-inflammatory heme oxygenase-1 (HO-1) into MI mouse model can rescue the function of left ventricular ejection fraction (LVEF) and decreased fibrosis, regardless of genetic modification. Besides, Guan et al. (2020) found that rats receiving iPSCs-CMs transplantation had lower mortality and fibrosis than the control group by improving left ventricular functional deterioration. These studies suggest that iPSCs-CMs have the potential to restore cardiac function by promoting myocardial repairment of infarcted myocardium and inhibiting the development of cardiac fibrosis.

#### 2.1.6 Endothelial cells

Endothelial cells help maintain cell barrier function and blood flow and are also one of the cells involved in inflammatory reactions. The phenotypic transformation from endothelial cells to mesenchymal cells which was later called “EndoMT” has been proven to contribute a lot to fibroblast accumulation in organ fibrosis (Piera-Velazquez et al., 2016). Previously reported that iPSCs-derived endothelial cell-based therapy could alleviate myocardial fibrosis and skin fibrosis. For example, Dai et al. (2011) showed that a tricell patch containing iPSCs-derived endothelial cells (iECs) could alleviate myocardial fibrosis in mouse MI model. There have been a small number of scientific reports on the therapeutic effect of iPSC on skin fibrosis such as hypertrophic scarring and systemic sclerosis (Azhdari et al., 2013; Ren et al., 2015). Azhdari et al. (2013) presented that skin fibrosis improved with a reduction in collagen content and the number of total and degranulated mast cells returned to their normal state after the transplantation of human iECs in bleomycin-induced systemic sclerosis mouse model. These researches indicate that iECs can be used to rescue organ fibrosis by replacing abnormal or impaired endothelial cells.

#### 2.1.7 The multicellular system consisting of iCMs and fibroblasts, and or iECs

Cardiac fibrosis is a complex disease that involves multiple cell types including CMs, endothelial cells, and fibroblasts (Nielsen et al., 2019). In addition to the loss of functional CMs and the phenotypic transformation of endothelial cells to mesenchymal cells in myocardial fibrosis, activated fibroblasts are one of the main cells that cause pathological fibrosis and heart failure (Jun and Lau, 2018; Zhang et al., 2019). Studies have been well established that iPSCs-derived CFs preserve a quiescent phenotype and are highly similar to primary CFs at transcriptional, cellular, and functional levels (Zhang et al., 2019; Zhang et al., 2020). Moreover, Zhang et al. (2019) identified that crosstalk between human iPSCS-CMs and CFs via the atrial/brain natriuretic peptide-natriuretic peptide receptor-1 (ANP/BNP-NPR1) pathway might represent a novel channel for anti-cardiac fibrosis therapy. As previously reported, a multicellular system consisting of iCMs and fibroblasts, and or iECs may be used to reduce myocardial fibrosis in infarcted hearts (Dai et al., 2011; Błyszczuk et al., 2020; Guan et al., 2021). For example, Dai et al. (2011) revealed that a tricell patch consisting of iCMs, iECs, and mouse embryonic fibroblasts affixed over the entire infarcted area reduced myocardial fibrosis and improved left ventricular function in the mouse MI model. Another study obtained three-dimensional (3D) cardiac microtissues including iCMs and human cardiac fibroblasts and then found that cardiac fibrosis in 3D microtissues was inhibited by treatment with the TGF-β1 inhibitor SD208 by a β-adrenoreceptor-dependent mechanism (Błyszczuk et al., 2020). Besides, the spleen acts as a hematopoietic stem cell niche and secretes cardioprotective factors after MI (Lieder et al., 2018). The splenic matrix can serve as a platform for stem cell culture and a cell carrier for transplantation (Crane et al., 2017). Previous studies have reported that injectable hydrogels could serve as effective carriers to deliver therapeutic cells and biomolecules through minimally invasive surgery, thus promoting tissue regeneration (Zhang et al., 2018; Feng et al., 2019). Guan et al. (2021) demonstrated that the application of thermoresponsive nanofibrous splenic matrix hydrogel (SpGel)-encapsulated iECs/iCMs effectively improved cardiac function and inhibited cardiac fibrosis in infarcted hearts. These studies indicate that after transplantation of a multicellular system *in vivo*, organ fibrosis can be improved to a certain extent by inactivating and eliminating myofibroblasts and replacing damaged CMs and or endothelial cells.

#### 2.1.8 Progenitor cells

Progenitor cells provide a potentially valuable pathway for organ repairment in organ fibrosis due to their multipotency and proliferative ability (Imberti et al., 2015; Toyohara et al., 2015; Xuan et al., 2018). Researchers used a cardiogenic small molecule isoxazole (ISX-9) to produce human cardiac progenitor cells (CPCs) from human iPSCs and then found that cell therapy for cardiac fibrosis with iPSCs-derived CPCs transplantation ameliorated fibrosis and improved heart function in immunodeficient mouse MI model (Xuan et al., 2018). Furthermore, Toyohara et al. (2015) found that renal subcapsular transplantation of human iPSCs-derived renal progenitor cells (RPCs) could improve acute kidney injury in mouse induced by ischemia/reperfusion injury, attenuating histopathological changes including tubular necrosis, tubule dilatation with casts and interstitial fibrosis. These studies suggest that iPSCs-derived CPCs have the potential to restore organ function by inhibiting tissue fibrosis.

### 2.2 iPSCs-derived extracellular vesicles, exosome, and conditioned media-based therapy for organ fibrosis

#### 2.2.1 Extracellular vesicles

Extracellular vesicles (EVs) are cell-derived membrane nanoparticles and one of its function is to mediate cell-to-cell communication by transferring biomolecules between cells (Diao et al., 2022). Some research emphasizes the role of stem cell-derived EVs in the healing of infarcted hearts and as potential antifibrotic agents (Khan et al., 2015; Povero et al., 2019; d'Alessandro et al., 2021). The activation of HSCs-the transformation of quiescent, vitamin A support cells into proliferative fibroblasts has been recognized as the central driver of liver fibrosis (Tsuchida and Friedman, 2017). Povero et al. (2019) revealed that the uptake of iPSCs-EV by hepatic stellate cells (HSCs) would lead to the reduction of fibrosis-promoting markers, the activation of HSCs, and the reduction of liver fibrosis. This study highlighted iPSCs-EVs as a novel antifibrotic approach that may reduce or reverse liver fibrosis in patients with chronic liver disease.

#### 2.2.2 Exosomes

Exosomes are cell-secreted nanovesicles of endosomal origin, some research indicates that iPSCs-exosomes may be a potential therapy for PF. Zhou et al. have found that iPSCs-exosomes inhibit M2-type macrophages by targeting TET1 to deliver miR-302a-3p thereby alleviating PF (Zhou et al., 2021). Tamò et al. (2018) found that the secretome of iPSCs reduces lung tissue fibrosis with bleomycin damage by partially altering macrophages and modulating their gene expression.

Besides, exosomes have also been shown to be a major contributor to the stem cell-mediated paracrine effects observed during cardiac repair (Fan et al., 2020). Exosomes secreted from iCMs (iCM-Ex) can be collected in large quantities *in vitro* and injected as a cell-free therapy for MI instead of live iCMs. Santoso et al. (2020) revealed that mouse treated with iCM-Ex marked heart improvement and reduced fibrosis after MI, which could provide a novel cell-free, patient-specific therapy for ischemic cardiomyopathy. But the beneficial constituents in exosomes secreted by iPSCs have not been fully established (Shamis et al., 2011). Studies demonstrated that iPSCs-exosomal microRNAs (miRNAs, miR) including miR-21 and miR-210 presented cardioprotective effects by enhancing CMs survival or proliferation and attenuating cardiac fibrosis (Moghaddam et al., 2019). Nevertheless, other studies have exhibited that exosomes secreted from human iPSCs-derived Mesenchymal stem cells (MSCs) increase their secretion of collagens and elastin (Hu et al., 2015; Zhang et al., 2015). Moreover, differential exosome production and cargo content have been observed when CMs were stimulated under various stress conditions (Jung et al., 2017). These differences may be due to the induced cell types and study conditions resulting in the production of different components in exosomes. The differences in the above findings require further studies to address these issues. Given the complexity of the molecules in these exosomes, great caution should be exercised in applying human iPSCs with mutant genotypes (Jung et al., 2017; Fan et al., 2020). So, the iPSCs-derived exosomes should be thoroughly studied to identify the beneficial and potentially harmful components before the routine clinical application of exosome-based therapy.

#### 2.2.3 Conditioned media

In recent years, stem cells and their secretomes have been investigated as novel treatments for PF. Gazdhar et al. (2014) collected iPSC-conditioned media (iPSC-cm) from cultured iPSCs derived from human foreskin fibroblasts. Their research showed iPSC-cm increased alveolar epithelial wound repair *in vitro* and attenuated bleomycin-induced fibrosis *in vivo*, which may be a promising novel, cell-free therapeutic option against lung injury and fibrosis. Further studies demonstrated that the improved function of iPSC-cm in PF may be achieved by blocking the role of the TGF-β1/*drosophila* mothers against the decapentaplegic (Smad) signal transduction pathway (Zhou et al., 2018).

Hypertrophic scarring (HS) is a type of fibrosis occurring in the skin and is characterized by fibroblast activation and excessive collagen production. Previous studies have shown that iPSC-cm may prevent the processes that lead to hypertrophic scar formation by attenuating fibroblast activation, blocking the recruitment and adhesion of inflammatory cells, and reducing the contractile capacity of fibroblasts (Ren et al., 2015). We believe that more studies will further explore iPSC applications in skin fibrosis in the future.

In summary, there has a homologous molecular mechanism underlying the anti-fibrotic action of iPSCs or their derivatives. For example, both undifferentiated iPSCs, iPSCs-derived AEC2, iCMs, and conditioned media transplantation could regulate fibrosis by inhibiting the TGF-β pathway. Given the general mechanism of fibrosis, iPSCs-derived epithelial including AEC2 or hepatocytes-based therapy can prevent organ fibrosis by reducing inflammation and reducing ECM protein. Furthermore, iPSCs-derived EVs, exosomes, and conditioned media-based therapy can also work for different organ fibrosis.

## 3 Gene-editing iPSCs in basic and translational discovery for organ fibrosis

Some fibrotic diseases including congenital hepatic fibrosis (CHF) and cystic fibrosis are closely related to genetic mutations. The gene-editing iPSCs may bring great hope to individualized or precision therapies for the above diseases. CHF is a rare inherited disorder caused by mutations in the PKHD1 gene with abnormal proliferation of cholangiocytes and variable degrees of periportal fibrosis (Tsunoda et al., 2019; Hasbaoui et al., 2021). Researchers knock out PKHD1 in iPSCs-derived hepatic progenitor-like cells to successfully establish a CHF disease model. And they revealed that the loss of fibrocystin encoded by PKHD1 triggers the proliferation of cholangiocytes through increased production of connective tissue growth factor (CTGF) and interleukin 8 (IL-8), moreover, IL-8 and CTGF levels were also significantly higher in the liver of patients with CHF which strongly indicated that CTGF and IL-8 will be novel therapeutic targets for the prevention of CHF (Tsunoda et al., 2019; Hasbaoui et al., 2021) (Supplementary Table S2).

Cystic fibrosis is a genetic disease caused by mutations in the cystic fibrosis transmembrane conductance regulator (CFTR) gene, which encodes a chloride channel that regulates the balance of salt and water in secretory epithelial cells (Ruan et al., 2019; Merkert et al., 2020). It is the most common life-limiting, autosomal recessive monogenic disease in Caucasian populations (Farrell et al., 2008; Cantin et al., 2015). CFTR encodes an ion channel that is mainly expressed in the secretory epithelial cells of the airway, intestine, liver, and other tissues (Engels et al., 2019). Although cystic fibrosis influences multiple organs, progressive airway remodelling, mucus accumulation, and chronic inflammation of the lung leading to respiratory failure are the major cause of morbidity and mortality (Nuciforo and Heim, 2021). Past studies have generated cell lines related to F508del mutation (Fleischer et al., 2018; Kondrateva et al., 2020; Kondrateva et al., 2021a; Kondrateva et al., 2021b), Asn1303Lys mutation (Merkert et al., 2020), and S308X mutation (Khor et al., 2022) with human iPSCs derived from cystic fibrosis patients. These iPSC lines derived from the somatic cells of cystic fibrosis patients provide useful resources for disease modelling and research on the pharmacological response to CFTR regulators. In particular, iPSC cell lines carrying nonsense mutations could bring therapeutic hope to patients with extremely rare mutation types. Besides, several studies have used CRISPR, zinc-finger nuclease-mediated homology-directed repair, small/short DNA fragments, and sequence-specific TALENs to precisely correct mutations responsible for cystic fibrosis in patient iPSCs-derived epithelial cells (Crane et al., 2015; Firth et al., 2015; Suzuki et al., 2016; Merkert et al., 2017). Previous research presented directional differentiation of human iPSCs into airway basal cells (iBCs), a population similar to airway epithelial stem cells. Hawkins et al. corrected the F508del CFTR mutation by gene editing and finally revealed that the correction of the F508del mutation resulted in the restoration of the CFTR-dependent current (Hawkins et al., 2021). These discoveries showed that these gene-editing iPSCs-derived airway epithelial may be applied to the disease models and promote the development of iPSC-based cell therapies (Firth et al., 2015; Suzuki et al., 2016; Hawkins et al., 2021) (Supplementary Table S2) In general, researchers use gene-editing iPSCs to create disease models that mimic the pathology of human diseases. By studying these models, researchers may gain insights into the mechanisms behind the disease and identify potential drug targets.

## 4 Comparison between iPSCs, ESCs, MSCs, and small-molecular drugs

PSCs including iPSCs and ESCs. All of the above cells can self-renew and differentiate into ectoderm, mesoderm, and endoderm derivatives. Human iPSCs used as alternative sources of PSCs have the same differentiation potential to transform into several lineages as ESCs. These properties make them very promising in drug screening, disease modeling, and regenerative medicine (Dvash et al., 2007). However, ESCs are derived from human embryos with limited supplements, and there are ethical issues related to embryos, which will limit the wide application of ESCs in clinical. But human iPSCs can be easily obtained by reprogramming patient-specific cells, which is suitable for mass production. By not involving embryos, some ethical issues can be avoided, and can be applied to personalized medicine. Moreover, ESCs also have safety problems related to immune rejection (Omole and Fakoya, 2018). The development of iPSCs reduces the risk of immune rejection because if the donor and the recipient are the same patients, immune rejection can be avoided (Nicolas et al., 2016; Guha et al., 2017). However, because iPSCs are reprogrammed from somatic cells, they may carry some undesirable mutations or epigenetic changes, while ESC is unlikely to have such problems (Liu et al., 2018; Parrotta et al., 2020). In general, human iPSCs may be the preferred choice because of their availability and lack of potential ethical concerns associated with human ESCs (Fan et al., 2020).

Mesenchymal stem cells (MSCs) are multipotent stromal progenitor cells that are heterogeneous, can self-renew, and transform into several lineages (Rastegar et al., 2010). MSCs can be obtained and isolated from a variety of tissues, such as bone marrow, skin, adipose tissue, umbilical cord blood, amniotic fluid, and placenta, of which bone marrow is the main source (Rastegar et al., 2010). Both the iPSCs and MSCs can easily be obtained and have the potential to differentiate into multiple lineages including osteoblastic, chondrogenic, adipogenic, neuronal, and cardiomyogenic lineages (Rastegar et al., 2010; Singh et al., 2015; Zomer et al., 2015). And both of them have a potential for potential tumorigenicity and the risk of chromosome aberration and genetic instability (Karnoub et al., 2007; Okita et al., 2008; Barkholt et al., 2013; Liu et al., 2018; Parrotta et al., 2020). Compared with the low reprogramming efficiency of iPSCs, the advantages of MSCs include that they can be produced on a larger scale and can be widely used in clinical applications such as neurodegenerative diseases, skin diseases, and liver diseases (Scudellari, 2016; Shin et al., 2017; Nasiri et al., 2019; Boika et al., 2020). Human iPSCs are stable at low temperatures without loss of function. Nevertheless, MSCs may experience replicative senescence, which may be related to the lack of telomerase activity, the decrease of cell proliferation of MSCs, and the reduction of differentiation potential of osteogenic and chondrogenic (Stenderup et al., 2003; Zhao et al., 2019; Fan et al., 2020). Furthermore, compared with MSCs, human iPSCs can be readily obtained by reprogramming patient-specific cells and avoiding immune rejection, because the donor and the recipient are the same patients, which may be used for precision therapy in organ fibrosis (Singh et al., 2015; Shi et al., 2017). The previous study demonstrated improved cardiac function with decreased fibrosis in both human iPSCs-CMs and human MSCs groups when compared with the MI group. And cardiac fibrosis was decreased in the human iPSCs-CM group when compared to the human MSCs (Citro et al., 2014). However, the exact mechanism of human iPSCs-CMs reducing cardiac fibrosis needs further study.

Ninety percent of patients with cystic fibrosis now benefit from small-molecule targeted therapies including Kalydeco (ivacaftor), Orkambi (lumacaftor/ivacaftor), Symdeko (tezacaftor/ivacaftor and ivacaftor) and Trikafta (elexacaftor/tezacaftor/ivacaftor and ivacaftor), but these treatments are not applied for those bearing nonsense mutations (Khor et al., 2022). Despite the relative success of symptomatic and pathological treatments, cystic fibrosis is incurable in most cases and patients must permanently take these costly drugs, which put significant financial stress on patients (Kondrateva et al., 2021a). The development of new approaches including iPSC-based gene therapy is becoming a highly hot issue now (Kondrateva et al., 2021a). The pluripotency and genomic profile of the iPSC cell lines have been validated as a resource that can be used for pharmacological investigations and drug screening associated with mutations of CFTR genes and provide hope for curing cystic fibrosis in the future (Merkert et al., 2020). Another advantage of iPSCs compared with small-molecular drugs is that the patient-specific iPSC cell lines carrying nonsense mutations could bring new therapeutic hope, including the development of personalized pathogenic therapy, pharmacological research, and precise medical drug screening for patients with extremely rare mutation types (Merkert et al., 2020). However, because iPSCs are obtained through gene reprogramming technology, there may be potential genetic mutations and epigenetic changes that may cause safety issues (Liu et al., 2018; Parrotta et al., 2020). Furthermore, the generation and identification of iPSC cell lines still require high costs, and the efficiency of reprogramming is generally low. Therefore, an economic and effective method for generating and differentiating iPSC is necessary for cell-based therapy and drug screening for personalized therapy.

## 5 Challenges and future outlook

Human iPSCs are characterized by human origin, pluripotency, and ultimate differentiation into any disease-related cell type, as well as epigenetic and genetic matching to the patients they have been derived from, which makes them a reliable avenue for organ fibrosis treatment. Human iPSCs are readily available, suitable for mass production, and stable at low temperatures without loss of function. Besides, iPSCs may eliminate ethical issues and religious concerns compared with ESCs. Moreover, human iPSCs may reduce the risk of immune rejection and be able to achieve personalized precision therapy by using patient-specific stem cells allowing for gene targeting and gene editing technology to correct mutations (Chun et al., 2011; Choi et al., 2013).

iPSC technology inevitably has some limitations associated with the reprogramming process: 1) Since iPSC is produced by retrovirus or lentivirus vectors, the genetic material introduced by retrovirus may be randomly integrated into the host genome, which may cause genetic abnormalities and teratoma (Singh et al., 2015). Recently, some of the safety problems concerning iPSC-based cell therapy and basic discovery have been partially resolved by using a no-integration virus, viral-free constructs, or even recombinant proteins. These methods make iPSCs safer and more suitable for clinical use (Okita et al., 2008; Stadtfeld et al., 2008; Yu et al., 2009; Zhou et al., 2009; Wysoczynski and Bolli, 2020). 2) The undifferentiated iPSCs have the risk for potential tumorigenicity. The iPSC under undifferentiated conditions may develop into teratoma and malignant tumors such as neuroblastoma and follicular carcinoma. There is also a risk of tumorigenesis after transplantation of contaminated iPSCs (Okita et al., 2008). The use of fluorescent-activated cell sorting or magnetic bead sorting purification can eliminate the potential tumorigenic risk to a certain extent (Sutermaster and Darling, 2019). 3) Reprogrammed cells may retain the epigenetic feature of the somatic cells where they originate, and the accumulation of chromosomal and/or mutations may display genomic instability (Liu et al., 2018; Parrotta et al., 2020). Therefore, before clinical application, these technologies should be tested in large animal models because they can predict human responses better than rodents (Harding et al., 2013; Nicolas et al., 2016). 4) More and more laboratories adopted iPSCs, however, only a small number of cells are eventually completely reprogrammed due to the inefficiency of reprogramming techniques, and there is heterogeneity among iPSC cells, therefore it is a big challenge to make every researcher under unified quality control (Scudellari, 2016). 5) Recent advances in gene-editing technologies, particularly CRISPR/Cas9, enable the rapid generation of disease models based on genetically defined human iPSC. But a major challenge in using the application of CRISPR/Cas9 technology is the possibility of off-target effects (Shi et al., 2017). Gene editing tools are also constantly being improved which may help to solve the problem of off-target effects. 6) Human iPSCs carry large amounts of private information (DNA), if abused, may violate the law and personal privacy. Despite the death of the donor, iPSCs still contain information about their close relatives, which may be an ethical and legal issue regarding personal privacy (Lin et al., 2004).

On the other hand, the limitations of iPSC reprogramming technology as mentioned above lead to similar deficiencies in iPSCs-derived cells. Furthermore, iPSCs-derived cells cannot be fully consistent with the phenotype and function of endogenous tissue-resident mature somatic cells (Zhang et al., 2009; Zhou et al., 2014; Toyohara et al., 2015; Choi et al., 2020; Tanaka et al., 2022). For example, previous studies have shown that even under standard two-dimensional culture conditions, after 8 weeks of iPSCs differentiation, the phenotype of mature cells derived from iPSCs is still in an embryonic state, but the 3D culture methodology can simulate the environment of mature somatic cell cells, which may promote cell differentiation and phenotype transformation to some extent (Zhang et al., 2009; Jacob et al., 2017). In addition, several studies reported that iPSCs-derived macrophages and monocyte-derived macrophages have similar phenotypes and functions. But the phenotype of iPSCs-derived macrophages is sometimes different from that of monocytes-derived macrophages or tissue-resident macrophages (Tanaka et al., 2022). Zhou et al. revealed that a limited number of undifferentiated iPSCs express alveolar epithelial markers during the culture process, indicating a lower efficiency of iPSC differentiation (Zhou et al., 2014). Besides, Toyohara et al. reported that the expression levels of some endothelial markers in human iPSCs-derived endothelial cells and natural human cardiac endothelial cells are different, which indicates that human iPSCs-derived endothelial cells may not be as mature and stable as mature somatic cells (Fan et al., 2022). However, the above limitations can be achieved by optimizing cell culture conditions and using ECM of normal mature somatic cell cells, which may improve the differentiation efficiency of iPSC and the ability to recapitulate the phenotype and function of mature somatic cell cells (Zhang et al., 2009; Chen et al., 2011).

Despite its functional role in the pathogenesis and treatment of fibrosis, the use of human iPSC-based cell therapy for organ fibrosis is still in the experimental stage after years of research. Problems include bioethical constraints, the safety of cell transplantation, routes of delivery, and the dose and timing of administration. Further studies are needed to establish the optimal strategy for the effective use of cell therapy for organ fibrosis (Ghadiri et al., 2016). Researchers must persevere and patiently conduct further research related to drug discovery based on iPSC. They will need strong support from the pharmaceutical industry and the government to advance cell therapy. The clinical application of iPSCs still has a long way to go.
