# Supplementary material for: Induced pluripotent stem cell-based therapies for organ fibrosis
Source: Front Bioeng Biotechnol. 2023 May 18;11:1119606. doi: 10.3389/fbioe.2023.1119606 (PMC10232908; doi:10.3389/fbioe.2023.1119606)
Supplement: Supplementary file 1 [file Table1.docx]

**Supplement Table S1. iPSCs-based therapeutic approaches for treating organ fibrosis in vivo**

| **iPSCs-derivatives** | **Types of fibrosis** | **Disease models** | **Treatments in vivo** | **Biological effects** | **Significances** | **Limitations** | **References** |
| --- | --- | --- | --- | --- | --- | --- | --- |
| three-gene iPSCs  (c-Myc-free) | PF | bleomycin-induced fibrosis mouse model | administrated intravenously iPSC 24h after bleomycin instillation | suppress inflammatory responses, TGF-β1/Smad 2/3 pathway, and EMT during the progression of PF | providing new useful clues regarding the mechanisms of iPS cells in the treatment of this disease | the tumorigenicity after iPSC transplantation has not been explored | (Zhou et al., 2016) |
| three-gene iPSCs  (c-Myc-free) | PF | bleomycin-induced fibrosis mouse model | intravenous delivery of three-gene iPSCs | alleviated PF by attenuating collagen content and neutrophil accumulation | alleviated the severity of histopathologic and physiologic impairment in lung fibrosis | the undifferentiated iPSCs may have the risk for potential tumorigenicity after 2 months of follow-up | (How et al., 2013) |
| macrophages | liver fibrosis | CCl_4_-induced toxic mouse model | Human iPSC-M1 or -M2(5x10^6^cells/mouse, 2 x IP injection) | iPSCs-derived macrophage populations reduce fibrogenic gene expression and disease-associated histological markers in immunodeficient mouse model | highlighting the potential of human iPSCs-derived macrophages as an anti-fibrotic liver therapy | since this study used an immunodeficient mouse model, the effect of endogenous immune cells including macrophages and neutrophils is less clear in this research and true human iPSCs-derived macrophages localized in the liver were not detected | (Pouyanfard et al., 2021) |
| alveolar epithelial cell | PF | bleomycin-induced fibrosis rat model | intratracheal transplantation of human iPSCs-derived AEC2 | reduced expression of α-SMA and TGF-β in the experimental bleomycin model rats | transplantation of human induced pluripotent differentiated to alveolar type II-like cells halts and reverses fibrosis | iPSC differentiated into AEC2 did not fully resemble endogenous lung AEC2 | (Alvarez-Palomo et al., 2020) |
|  | PF | bleomycin-induced fibrosis mouse model | intratracheal transplanted hiPSCs-derived ATIICs | remain and re-epithelialize injured alveoli to restore pulmonary function | allows for the first-time efficient generation of patient-specific ATIICs for possible future clinical applications | the mechanism of a low dose of G418 used for ATIIC selection, which allows more efficient production of human iPSC-ATIICs is still unknown | (Yan et al., 2014) |
|  | PF | bleomycin-induced fibrosis mouse model | intratracheal transplantation of hiPSCs-derived AECs | reduces lung inflammation and attenuates lung fibrosis | represent new opportunities in lung tissue engineering and cell therapy | the efficiency of iPSC differentiation to lung epithelial cells by the lung scaffold is low | (Zhou et al., 2014) |
| hepatocytes | liver fibrosis | copper-induced hepatocyte toxicity mouse model | cellular transplantation of ATP7B iHeps into mouse via intra-splenic injection | liver function improved and fibrosis decreased due to reductions in hepatic copper accumulation and copper-induced hepatocyte toxicity | iHeps derived from gene-corrected WD iPSCs have potential use as an autologous ex vivo cell source for the therapy of WD | the influence of iHep therapy was uncertain for WD patients with mainly neurological impairment | (Wei et al., 2022) |
|  | liver fibrosis | thioacetamide-induced mouse model | intravenously transplantation of iHep cells | decreased liver fibrosis, apoptotic cells in the liver, and ameliorated abnormal liver function | the liver regeneration potential of genome-edited iHep cells and for further studies exploring cell therapy as an alternative therapeutic option for the treatment of liver fibrosis | a better cell differentiation protocol for the generation of more specific hepatic cells from iPSCs is needed | (Choi et al., 2020) |
| hepatocyte-like cells | liver fibrosis | CCl_4-_induced liver injury mouse model | iPS-HLCs (1×10^6^ cells/mouse) were intrasplenically transplanted | the survival rate of the acute liver injury mouse increased and the liver fibrosis decreased | maybe generate safe and therapeutically effective human iPS-HLCs for hepatocyte transplantation. | a teratoma formation assay should be performed in the subcutaneous space and liver | (Takayama et al., 2017) |
| cardiomyocytes | cardiac fibrosis | porcine ischemic cardiomyopathy model | transplant cardiac tissues developed by culturing iPSCs-derived CMs | interstitial fibrosis attenuated, hypertrophied cardiomyocytes attenuated, and capillary density increased | improved cardiac function with angiogenesis and antifibrotic effects in a porcine cardiomyopathy model | the benefits of iPSCs-derived CMs were not fully achieved in experiments because of insufficient engraftment | (Suzuki et al., 2021) |
|  | cardiac fibrosis | MI rat model | injected hAFSC-iPSC-CMs intramuscularly into rat-infarcted hearts | restored cardiac function, partially remuscularized the injured region, and reduced fibrosis in the rat-infarcted hearts | hAFSC-iPSCs are potential candidates for the repair of infarcted myocardium | the group numbers of animal studies are limited; did not provide the proliferation and apoptosis information at post-transplant 24 hours | (Fang et al., 2020) |
|  | cardiac fibrosis | MI mouse model | inject genetically modified hiPSC-CMs into the hearts of the more receptive strain | administration of hiPSC-CMs increased LVEF after MI and decreased fibrosis | hiPSC-CMs are effective in improving heart function after MI | not address the role of immune cells in hiPSC-CM-based cell therapy outcomes | (Stępniewski et al., 2020) |
|  | cardiac fibrosis | MI mouse model | transplanted iPSCs-derived CMs in the infarcted mouse heart | inhibited apoptosis and fibrosis and improved cardiac function compared with MI and MI+H9c2 cell groups | contain the potential to differentiate into CMs in the cell culture system and repair and regenerate infarcted myocardium with improved cardiac function in vivo | the exact reason for the absence of teratoma formation is completely unknown | (Singla et al., 2011) |
|  | cardiac fibrosis | postinfarction heart failure rat model | intramyocardial injection of iPSC-CMs | the grafted CMs survived in the rat heart 1 month after iPSC-CMs transplantation; myocardial fibrosis attenuated in the iPSC-CMs treatment group | cell replacement therapy reversed ventricular remodeling, indicating the potential of iPSC-CMs for cardiac repair strategies | MRI was not performed in the heart failure rat model; the use of hiPSC-CMs to treat immunocompetent rats may induce severe rejection caused by species differences | (Guan et al., 2020) |
|  | cardiac fibrosis | MI rat model | injected hiPSC-CMs into the ischemic heart 30-min after LAD ligation | cardiac fibrosis decreased in the hiPSC-CMs group compared with hMSCs or MI groups | improved cardiac function with decreased fibrosis with both hiPSC-CMs and hMSCs groups when compared with the MI group | the beneficial factors released by hiPSC-CMs compared to hMSCs need further investigation | (Citro et al., 2014) |
| endothelial cells | cardiac fibrosis | MI mouse model | application of SpGel- encapsulated iEC/iCMs | improved cell graft retention accelerated cardiac function recovery inhibited fibrosis and promoted revascularization of ischemic tissue | established a novel platform for the generation and delivery of autologous cell grafts, which could be a promising clinical therapeutic strategy for cardiac repair and regeneration after MI | the mechanisms of functional improvement in cardiac differentiation and cardiac repair mechanisms of SpGel need to be elucidated | (Guan et al., 2021) |
|  | skin fibrosis | bleomycin-induced systemic sclerosis mouse model | transplantation of human iPSC-endothelial cells | a reduction in collagen content and the number of total and degranulated mast cells returned to their normal state | maybe used as an appropriate source for the treatment of systemic sclerosis patients | limitation in the expansion potential of hiPSC-ECs; require more reliable cell tracking, noninvasive cell tracking, and immunodeficient mice | (Azhdari et al., 2013) |
| tricell patch | cardiac fibrosis | MI mouse model | affixed Tri-P over the entire infarcted area 7 days after MI in mouse overexpressing AC6 | LV function improved progressively in AC6 mouse from weeks 2 to 4 and was associated with reduced LV fibrosis. | Application of a Tri-P in AC6 mouse resulted in higher iPSC engraftment accompanied by angiomyogenesis in the infarcted area and improvement in LV function. | the surgical procedure for cell patch transplantation is invasive and requires thoracotomy | (Dai et al., 2011) |
| cardiac progenitor cells | cardiac fibrosis | MI mouse model | intramyocardial injection of CPCs | CPCs transplantation promoted neoangiogenesis, attenuated fibrosis, and led to functional improvement | found a novel strategy for human CPCs generation from hiPSCs using a cardiogenic small molecule, isoxazole (ISX-9) | strategies of CPCs derived from hiPSCs are labor-intensive and time-consuming, with high production costs, which limit the clinical application | (Xuan et al., 2018) |
| renal progenitor cells | kidney fibrosis | ischemic acute kidney injury mouse model | renal subcapsular transplantation of human iPSCs-derived RPCs | improved acute kidney injury in mouse induced by ischemia/reperfusion injury, attenuating histopathological changes including tubular necrosis, tubule dilatation with casts, and interstitial fibrosis | regenerative medicine strategies for kidney diseases could be developed using hiPSCs-derived renal cells | hiPSC-derived cells generated in the study had limited developmental competence as RPCs because they differentiated mainly into proximal tubular cells | (Toyohara et al., 2015) |
| Extracellular vesicles | liver fibrosis | CCl4 and bile duct ligation-induced liver injury mouse model | intravenous injection of iPSC-EVs | uptake of iPSC-EV by HSCs would lead to the reduction of liver fibrosis | iPSC-EVs as a novel antifibrotic approach that may reduce liver fibrosis in patients with chronic liver disease | the study did not demonstrate the Evs' cellular localization after they accumulate in the liver | (Povero et al., 2019) |
| Exosomes | PF | bleomycin-induced fibrosis mouse model | intratracheal instillation of exosomes | iPSC-exosomes inhibit M2-type macrophages thereby reducing PF | iPSC-exosomes may become a potential therapeutic agent for pulmonary fibrosis | iPSCs-derived exosomes should be thoroughly studied to identify the beneficial and potentially harmful components | (Zhou et al., 2021) |
|  | cardiac fibrosis | MI mouse model | intramyocardial injection of exosomes | MI mouse treated with iCM-Exosomes marked heart improvement and reduced fibrosis | provide a novel cell-free, patient-specific therapy for ischemic cardiomyopathy |  | (Santoso et al., 2020) |
| Conditioned media | PF | bleomycin-induced fibrosis mouse model | intratracheal instillation of iPSC-cm | iPSC-cm attenuated bleomycin-induced fibrosis | iPSC-cm may be a promising novel, cell-free therapeutic option against lung injury and fibrosis | more detailed research on the secretome of iPSCs still needs to be elucidated | (Gazdhar et al., 2014) |

**Abbreviations**: iPSCs, induced pluripotent stem cells; hiPSCs, human induced pluripotent stem cells; iPS, induced pluripotent stem; AEC, alveolar epithelial cell; AEC2, alveolar epithelial type 2 cell; iAEC2s, iPSCs after differentiation into AEC2s; ILD, interstitial lung disease; IPF, Idiopathic pulmonary fibrosis; PF, pulmonary fibrosis; α-SMA, α-smooth muscle actin; ATII, alveolar type II; EMT, epithelial-mesenchymal transition; three-gene iPSCs, iPSCs lacking the reprogramming factor c-Myc; CMs, cardiomyocytes; iCMs, iPSC derived cardiomyocytes; ONO, ONO-1301, Cs, camostat mesilate, Pf, pirfenidone; ECM, extracellular matrix; iECs, iPSC derived endothelial cells; SpGel, thermoresponsive hydrogel; MI, myocardial infarction; PCs, pericytes; MIF, myocardial interstitial fibrosis; hAFSCs, human amniotic fluid-derived stem cells; CPCs, cardiac progenitor cells; LAD, left-anterior-descending; MRI, magnetic resonance imaging; hMSCs, human mesenchymal stem cells; Tri-P, tricell patch; AC6, adenylyl cyclase 6; LV, left ventricular; LVEF, left ventricular ejection fraction; LAD, left anterior descending; iHeps, iPSCs-derived hepatocytes; HLCs, hepatocyte-like cells; RPCs, renal progenitor cells; WD, Wilson’s disease; EVs, Extracellular vesicles; HSCs, hepatic stellate cells; iPSC-cm, iPSC-conditioned media.

**Supplement Table S2. Gene-editing iPSCs in basic and translational discovery for organ fibrosis in vitro**

| **iPSCs-derived cell** | **Modeling method** | **Biological effect** | **Significance** | **References** |
| --- | --- | --- | --- | --- |
| cholangiocyte-like cells in cysts in a 3D-culture system | established PKHD1-knockout and heterozygously mutated PKHD1 iPS clones model of CHF | loss of fibrocystin function promotes the proliferation of cholangiocytes and the production of CTGF in an interleukin 8-dependent manner | IL-8 and CTGF are essential for the pathogenesis of CHF | (Tsunoda et al., 2019) |
| airway basal cells | used gene editing to correct the F508del CFTR mutation | correction of the F508del mutation led to the restoration of CFTR-dependent current | iPSCs-derived airway epithelium shares key physiologic and biological features with human airway epithelium | (Hawkins et al., 2021) |
| airway epithelial cells | using CRISPR to target corrective sequences to the endogenous CFTR genomic locus | normal CFTR expression and function were recovered | This isogenic iPSC-based model system for CF could develop new therapy | (Firth et al., 2015) |
| epithelial cells | used ZFN-mediated HDR to edit the endogenous CFTR locus and precisely correct mutations responsible for CF in patient-derived iPSCs | expressed the corrected CFTR gene; restored expression of the mature CFTR glycoprotein and restoration of CFTR chloride channel function in iPSCs-derived epithelial cells | using chamber assay in iPSCs-derived polarized epithelial monolayers should be a valuable tool in CFTR drug screening and analysis of various CFTR mutations | (Crane et al., 2015) |
| endoderm/airway-like epithelial cells | The F508del mutation in airway epithelial cell-derived CF-iPSCs was corrected with small/short DNA fragments (SDFs) and sequence-specific TALENs | TALENs Facilitate Single-step Seamless SDF Correction of F508del CFTR in Airway Epithelial Submucosal Gland Cell-derived CF-iPSCs | a corrected CF-iPSCs can be “differentiated” into airway epithelial-like cells through co-culture with immortalized AECs | (Suzuki et al., 2016b) |
| CFTR knock-in reporter cell line | - | optimized the differentiation of iPSCs into CFTR expressing epithelia on a single-cell level | represent an excellent tool for cystic fibrosis disease modelling, drug screening, and ultimately cellular therapies | (Engels et al., 2019) |
| iPSC line | derived from the cells of a 22-year-old woman with genetically proven CF (F508del mutation) | the analysis confirmed the origin of the cell line from the fibroblasts of the indicated patient | - | (Kondrateva et al., 2021b) |
|  | obtained skin fibroblasts with clinically manifested and genetically proven (F508del/W1282X) cystic fibrosis were successfully transformed into iPSCs | two iPSC lines showed a normal karyo type, expressed pluripotency markers, and exhibited the potential to differentiate into three germ layers | used for the development of personalized etiotropic therapy, disease modelling, cell differentiation and organoid formation, pharmacological investigations, and drug screening | (Kondrateva et al., 2021a) |
|  | generated an iPSC line from a CF patient homozygous for the p.Asn1303Lys mutation | - | a useful resource for disease modeling and investigating the pharmacological response to CFTR | (Merkert et al., 2020) |
|  | generated from two different p.F508del homozygous female CF patients’ somatic cells (skin fibroblasts and keratinocytes) | - | The lines carry the p.F508del mutation, have a normal karyotype, express pluripotency markers, and can differentiate into the three germ layers | (Fleischer et al., 2018) |
|  | used TALENs and single-stranded oligonucleotides to correct the mutated triplet in our CF-iPSC line | - | - | (Merkert et al., 2017) |

**Abbreviations**: iPSC, induced pluripotent stem cell; iPSCs, induced pluripotent stem cells; iPS, induced pluripotent stem; CHF, Congenital hepatic fibrosis; CTGF, connective tissue growth factor; CF, cystic fibrosis; CFTR, Cystic Fibrosis Transmembrane Conductance Regulator; 3D, three-dimensional; ZFN, zinc-finger nuclease; HDR, homology-directed repair.
